# Kv4.2 channel activity controls intrinsic firing dynamics of arcuate kisspeptin neurons

**DOI:** 10.1113/JP274474

**Published:** 2018-01-11

**Authors:** Philipe R. F. Mendonça, Victoria Kyle, Shel‐Hwa Yeo, William H. Colledge, Hugh P. C. Robinson

**Affiliations:** ^1^ Department of Physiology, Development and Neuroscience University of Cambridge Downing Street Cambridge CB2 3EG UK

**Keywords:** firing irregularity, hypothalamus, kisspeptin, dynamic‐clamp, conductance injection

## Abstract

**Key points:**

- Neurons in the hypothalamus of the brain which secrete the peptide kisspeptin are important regulators of reproduction, and normal reproductive development.
- Electrical activity, in the form of action potentials, or spikes, leads to secretion of peptides and neurotransmitters, influencing the activity of downstream neurons; in kisspeptin neurons, this activity is highly irregular, but the mechanism of this is not known.
- In this study, we show that irregularity depends on the presence of a particular type of potassium ion channel in the membrane, which opens transiently in response to electrical excitation.
- The results contribute to understanding how kisspeptin neurons generate and time their membrane potential spikes, and how reliable this process is.
- Improved understanding of the activity of kisspeptin neurons, and how it shapes their secretion of peptides, is expected to lead to better treatment for reproductive dysfunction and disorders of reproductive development.

**Abstract:**

Kisspeptin neurons in the hypothalamus are critically involved in reproductive function, via their effect on GnRH neuron activity and consequent gonadotropin release. Kisspeptin neurons show an intrinsic irregularity of firing, but the mechanism of this remains unclear. To address this, we carried out targeted whole‐cell patch‐clamp recordings of kisspeptin neurons in the arcuate nucleus (Kiss1^Arc^), in brain slices isolated from adult male Kiss‐Cre:tdTomato mice. Cells fired irregularly in response to constant current stimuli, with a wide range of spike time variability, and prominent subthreshold voltage fluctuations. In voltage clamp, both a persistent sodium (NaP) current and a fast transient (A‐type) potassium current were apparent, activating at potentials just below the threshold for spiking. These currents have also previously been described in irregular‐spiking cortical interneurons, in which the A‐type current, mediated by Kv4 channels, interacts with NaP current to generate complex dynamics of the membrane potential, and irregular firing. In Kiss1^Arc^ neurons, A‐type current was blocked by phrixotoxin, a specific blocker of Kv4.2/4.3 channels, and consistent expression of Kv4.2 transcripts was detected by single‐cell RT‐PCR. In addition, firing irregularity was correlated to the density of A‐type current in the membrane. Using conductance injection, we demonstrated that adding Kv4‐like potassium conductance (*g_Kv4_*) to a cell produces a striking increase in firing irregularity, and excitability is reduced, while subtracting *g_Kv4_* has the opposite effects. Thus, we propose that Kv4 interacting dynamically with NaP is a key determinant of the irregular firing behaviour of Kiss1^Arc^ neurons, shaping their physiological function in gonadotropin release.

## Key points

- Neurons in the hypothalamus of the brain which secrete the peptide kisspeptin are important regulators of reproduction, and normal reproductive development.
- Electrical activity, in the form of action potentials, or spikes, leads to secretion of peptides and neurotransmitters, influencing the activity of downstream neurons; in kisspeptin neurons, this activity is highly irregular, but the mechanism of this is not known.
- In this study, we show that irregularity depends on the presence of a particular type of potassium ion channel in the membrane, which opens transiently in response to electrical excitation.
- The results contribute to understanding how kisspeptin neurons generate and time their membrane potential spikes, and how reliable this process is.
- Improved understanding of the activity of kisspeptin neurons, and how it shapes their secretion of peptides, is expected to lead to better treatment for reproductive dysfunction and disorders of reproductive development.

## Introduction

Hypothalamic kisspeptin‐GPR54 signalling is critical in regulating reproductive functions in mammals (d'Anglemont de Tassigny & Colledge, 2010). Disruption of this pathway leads to infertility in both sexes, as it prevents maturation of the gonads during puberty (de Roux *et al*. 2003; Seminara *et al*. 2003; Kirilov *et al*. 2013). The neuropeptide kisspeptin is produced in two distinct regions in the rodent hypothalamus: one localised in the rostral periventricular area of the third ventricle (Kiss1^RP3V^) and another in the arcuate nucleus (Kiss1^Arc^). The release of kisspeptin from these neurons is able to activate the G‐protein coupled receptor KISS1R (also known as GPR54), expressed by gonadotropin‐releasing hormone (GnRH) neurons (Han *et al*. 2005; Piet *et al*. 2014), which in turn stimulates GnRH neuron firing and causes the secretion of GnRH into the hypophyseal‐portal blood system.

Although both groups of kisspeptin neurons appear to synapse directly onto GnRH neurons, they show distinct properties. Notably, Kiss1^RP3V^ neurons synapse onto GnRH neuron cell bodies and proximal dendrites, and exhibit a sexual dimorphism, where the females have a larger neuronal population (Clarkson & Herbison 2006; Kauffman *et al*. 2007). In contrast, Kiss1^Arc^ neurons, whose population is relatively similar in size in both sexes (Clarkson & Herbison, 2006; Hoong Yip *et al*. 2015), synapse onto the more distal projections of the GnRH neurons within the median eminence region.

It is not entirely clear how these two populations regulate GnRH neuron firing. Recently, optogenetic stimulation was used to investigate how these kisspeptin neurons might integrate their inputs, and to test the firing conditions required for release of luteinizing hormone (LH) into the bloodstream. Firstly, optogenetic activation of GnRH neurons revealed that these cells require stimulation for at least 2 min at 10 Hz to evoke an LH surge (Campos & Herbison, 2014). Secondly, the excitation of GnRH neurons by upstream neurons was addressed (Han *et al*. 2015), revealing that Kiss1^Arc^ neurons also need to fire for 2–5 min at 10 Hz in order to increase LH in the blood to physiologically‐effective levels, via a kisspeptin/GPR54 mediated mechanism. Thirdly, the synaptic pathway through which Kiss1^Arc^ neurons recruit other kisspeptin neurons has also recently been uncovered (Qiu *et al*. 2016). Briefly, Kiss1^Arc^ neurons were shown to display a frequency‐dependent differential release of peptides (kisspeptin, neurokinin‐B and dynorphin) and amino acid neurotransmitters (glutamate), which, at high firing frequency (20 Hz), evoked synchronised activity of Kiss1^RP3V^ neurons, as well as ipsilateral and contralateral Kiss1^Arc^ neurons.

Although the circuitry in which kisspeptin neurons are embedded is slowly being unravelled, characterisation of the intrinsic electrophysiological properties of these cells remains incomplete. Several studies have shown that a variety of ion channels are present in these cells (Kelly *et al*. 2013; Zhang *et al*. 2013; Piet *et al*. 2014), but it is still unclear how the distribution and interaction of ion channels determines their spiking patterns and overall excitability.

In this paper, we have used a Kiss‐Cre:tdTomato transgenic mouse (Yeo *et al*. 2016) to further characterise the electrical properties of Kiss1^Arc^ neurons in males. Using patch clamp recording combined with conductance injection, we show that these cells have firing properties strongly influenced by fast inactivating potassium currents (A‐type current). This current imposes sub‐threshold membrane oscillations, which by interacting with other subthreshold currents – like persistent sodium currents (NaP) – generates spiking irregularity, even in the absence of synaptic activity. By characterising the A‐type current through means of pharmacological blockade and kinetics, combined with single‐cell RT‐PCR, it is demonstrated that this current is mediated mainly by Kv4.2. Using conductance injection, we were able to effectively control spiking irregularity and excitability in Kiss1^Arc^ neurons by modulating the level of Kv4‐type conductance. These results identify an important determinant of the firing dynamics of Kiss1^Arc^ neurons. The heterogeneous levels of Kv4 conductance present in these cells not only diversify their spiking patterns, but also control their excitability, suggesting that it might be a strong candidate for modulation in order to regulate Kiss1^Arc^ excitation onto GnRH neurons and other kisspeptin neurons.

## Methods

### Ethical approval

Animals were killed in accordance with the UK Home Office regulations under the Animal (Scientific Procedures) Act of 1986. The establishment, breeding and care of the mouse line were approved by a Local Ethics Committee at the University of Cambridge and performed under authority of a Home Office Licence (UK).

### Animal model

Arcuate kisspeptin neurons were located using a recently developed Kiss‐CRE:tdTomato mouse line (Yeo *et al*. 2016). Briefly, Cre recombinase was inserted in exon 2 after the first five amino‐acids of the KISS1 protein sequence (Met‐Ile‐Ser‐Met‐Ala). Next, the line was crossed with a reporter line expressing tdTomato under the constitutive CAG promoter, but preceded by a floxed STOP codon (Jackson Laboratories, Bar Harbor, ME, USA). The resulting offspring selectively expressed the tdTomato reporter only in *Kiss1*‐expressing cells. Since the Cre recombinase gene disrupts the *Kiss1* coding sequence, Kiss‐Cre homozygous mutant mice had impaired gonad development and thus were infertile. Heterozygous animals, which were all genotyped, show normal fertility and were used for breeding and for experiments.

**Figure 1:**
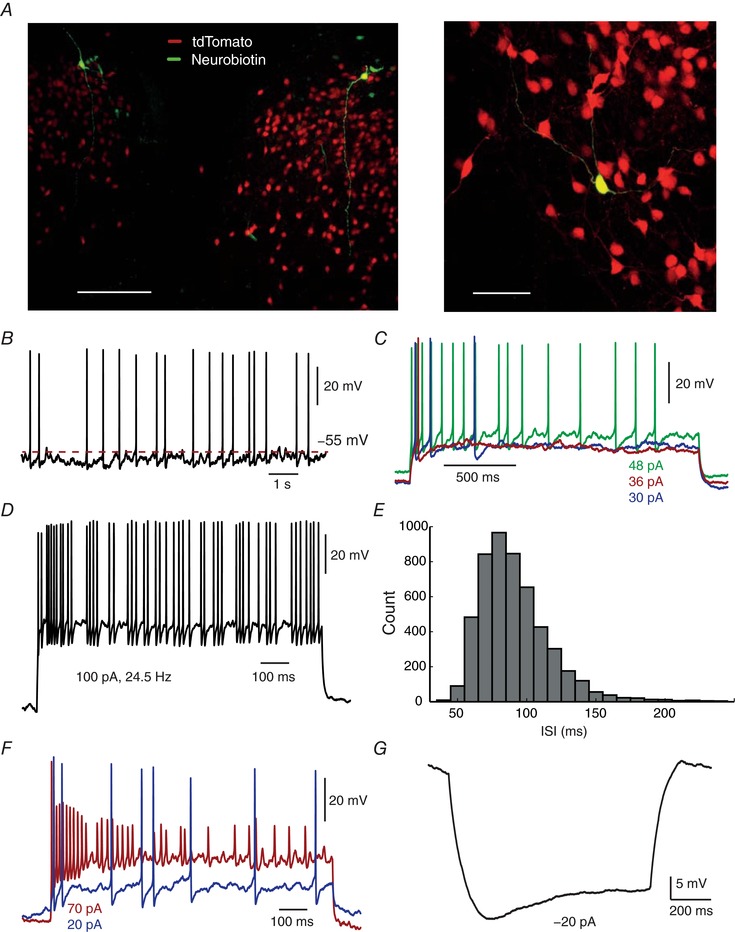
General electrophysiological properties of arcuate kisspeptin neurons *A*, left: distribution of Kiss1‐tdTomato cells in the arcuate nucleus in coronal sections, divided by the third ventricle. Right: detailed morphology of a neurobiotin‐filled neuron. Scale bars of 150 μm and 50 μm respectively. *B*, around 22% of the recorded cells displayed spontaneous firing, even in the presence of synaptic blockers. *C*, when stimulated with a square step current, most cells displayed a clear irregular‐spiking pattern, noticeably at lower firing frequencies. *D*, in other neurons, spiking irregularity was also present at higher firing frequencies. *E*, distribution of 5104 interspike intervals from a cell firing at 7–13 Hz. The broad distribution shows no distinct periodic peaks. Kurtosis: 8.28 Skewness: 1.60. *F*, kisspeptin neurons resisted firing at frequencies higher than approximately 40 Hz, as strong excitation led to depolarisation block. *G*, hyperpolarisation induced current (H‐current) observed in a fraction of kisspeptin neurons (14/49; seen here as a depolarising ‘sag’).

All data shown are from heterozygous mice, with the exception of the cells used for neurobiotin‐filled images (Fig. 1 *A*), for which we used homozygous mutant mice, as strong tdTomato expression in fibres allowed clearer confocal images. In order to exclude the physiological effects of the fluctuating levels of oestrogen throughout the oestrous cycle, only adult males were used for experiments.

### Brain slice preparation

Animals were killed by cervical dislocation, followed by quick opening of the skull and careful removal of the brain, which was placed in ice‐cold solution consisting of (in mm): 254 sucrose, 25 NaHCO_3_, 2.5 KCl, 1.25 NaH_2_PO_4_, 2 CaCl_2_, 1 MgCl_2_ and 10 glucose, maintained at pH 7.4 by bubbling with 95% oxygen/5% CO_2_ gas mixture (carbogen). Coronal slices 300 μm thick were obtained with a vibratome (LEICA VT1200S), and kept at room temperature for 40 min in artificial cerebrospinal fluid solution (aCSF), consisting of (in mm): 125 NaCl, 25 NaHCO_3_, 2.5 KCl, 1.25 NaH_2_PO_4_, 2 CaCl_2_, 1 MgCl_2_, 0.01 glycine and 25 glucose, equilibrated with carbogen.

Brain slices were imaged using an upright microscope (BX50WI) and perfused with carbogen‐equilibrated aCSF, heated to 30–33°C. Arcuate kisspeptin neurons were visualised with differential interference contrast (DIC) and identified by tdTomato epifluorescence.

### Patch‐clamp recordings

Electrophysiological recordings were carried out with an Axon Multiclamp 700B amplifier and CV‐7B headstage (Molecular Devices, Sunnyvale, CA, USA). Custom written Matlab software (Mathworks, Natick, MA, USA) was used to control a 16‐bit National Instruments X‐series board, with sampling and waveform generation at 20 KHz. Inputs were filtered before sampling using the built‐in 4 KHz Bessel filtering of the Multiclamp. The Multiclamp Commander software was used to obtain a bridge balance (current‐clamp experiments) and series resistance compensation (voltage‐clamp experiments; <30 MΩ and compensated up to 90%). Borosilicate glass capillaries (5–7 M Ω, Harvard Apparatus, Kent, UK) were used to pull patch pipettes with a PP‐83 puller (Narishige, Tokyo, Japan), which were then fire‐polished (Micro Forge MF‐830, Narishige) to promote high‐quality seal formation.

Passive properties were determined by applying a hyperpolarising step current (from −20 to −40 pA) in current‐clamp mode, and fitting an exponential curve to the response:

Vm(t)=IRe−t/τ+V rest ,

where, *R* is the input resistance, *I* is the step current injected, τ is the membrane time constant and *V*_rest_ is the resting membrane potential.

Firing properties were assessed in current‐clamp configuration, where synaptic activity was reduced by 10 μm gabazine, 10 μm CNQX and 10 μm APV. Firing irregularity was quantified by applying a square current step (2–10 s) sufficient to evoke firing at 7–13 Hz. Next, the interspike intervals (ISI) were used to calculate the coefficient of variation of the interspike interval (CVISI), defined as the ratio of the standard deviation of the ISI to the mean ISI.

Voltage‐gated potassium currents were analysed in the presence of tetrodotoxin (TTX, 300 nm). In both current and voltage‐clamp experiments, the intracellular solution used consisted of (in mm): 105 potassium gluconate, 30 KCl, 10 HEPES, phosphocreatine Na_2_, 4 ATP‐Mg, 0.3 GTP‐Na and 1 EGTA, balanced to pH 7.3 with KOH (10 mV liquid junction potential correction applied, 290–300 mOsm). Persistent sodium current was characterised with 2 mm 4‐AP, 2 mm TEA and 200 μm Cd^2+^ added to the extracellular solution, using an intracellular solution consisting of (in mm): 90 caesium methanesulfonate, 30 CsCl, 10 BAPTA, 10 HEPES, balanced to pH 7.3 with HCl (liquid junction potential = 12 mV). Salts were purchased from Sigma‐Aldrich (Dorset, UK), while synaptic blockers were acquired from Tocris Bioscience (Bristol, UK), and phrixotoxin from Abcam (Cambridge, UK).

### Artificial conductance injection

Kv4‐type conductance was artificially modulated in Kiss1^Arc^ neurons using the dynamic‐clamp technique (Robinson & Kawai, 1993; Sharp *et al*. 1993a; Robinson, 2013). In summary, a hard real‐time SM2 system (Cambridge Conductance; see Robinson, 2008) was used to sample the membrane potential and inject a fast‐inactivating outward conductance according to the Hodgkin‐Huxley‐type equation:

I Kv 4(t)=g¯mh(Vm−EK),

where $\bar{g}$ is the maximum conductance; *V*_m_ is the membrane potential; *E*_K_ is the K^+^ reversal potential; and *m* and *h* are the activation and inactivation variables, respectively, which obey:

$$\;\frac{dm}{dt}=\frac{m_{\infty }-m}{\tau_{m}}\;$$

and

$$\;\frac{dh}{dt}=\frac{h_{\infty }-h}{\tau_{h}}\;,$$

where *m_∞_* and *h_∞_* are the voltage‐dependent, steady‐state limiting values of *m* and *h*; and τ_m_ and τ_h_ are the voltage‐dependent time constant of activation and inactivation, respectively. The voltage‐dependent quantities *m_∞_*, *h_∞_*, τ_m_ and τ_h_ were specified as follows:

m∞(V)=11+ exp ((−30−V)/10) and τm(V)=0.346e−V/18.272+2.09,

h∞(V)=11+ exp (0.0878(V+55.1)) and τh(V)=2.1e−V/21.2+4.627.

The Kv4 conductance kinetics were determined from currents previously recorded in mouse cortical interneurons (Mendonça *et al*. 2016), which displayed virtually identical kinetics to those observed in Kiss1^Arc^ neurons. To facilitate interpretation of dynamic‐clamp experiments, the amount of artificial Kv4‐type conductance (*g*_Kv4_) injected is characterised by its peak value at 0 mV (*g*_max_, 0 mV).

### Confocal imaging

In some cases, neurobiotin (0.5%) was added to the intracellular solution in order to analyse the morphology of Kiss1^Arc^ neurons. Cells were patched for at least 20 min in order to allow complete diffusion of neurobiotin into finer processes of the neuron. Next, the slices were fixed in 4% paraformaldehyde overnight, followed by 3 × 15 min phosphate‐buffered saline (PBS) washes. Alexa 405 Fluor Streptavidin conjugated dye (1‐2 μg mL^−1^, Life Technologies) was then applied to the slice for 3 h at 5°C. Another series of PBS washes was done before mounting the samples on microscope slides with ProLong Gold antifade reagent (Invitrogen), which were then kept in the dark at 4°C for at least one day before imaging. Confocal images were acquired with a Leica SP2 microscope, and a minimum 2 μm resolution *z*‐stack was acquired for each cell.

### Cytoplasmic harvesting and single cell reverse‐transcription PCR

Single cell cytoplasm collection was carried out using a similar approach to that described previously (Yano *et al*. 2006; Subkhankulova *et al*. 2010). In summary, using an intracellular solution containing an RNAse inhibitor (RNase OUT, 1 U μL^−1^, Life Technologies), a standard whole‐cell recording mode was established. Next, a gentle negative pressure was applied (<150 mbar), creating a slow inward flow of the cytoplasm into the patch pipette. Proper care was taken to maintain the seal resistance. After approximately 2 min, most of the cytoplasmic content was collected, whereupon a slightly stronger pressure was applied in order to fully aspirate the nucleus. The contents of the pipette were then expelled into PCR tubes containing random primers, oligo‐dT primers, dNTPs (Promega, Southampton, UK), RNaseOUT (Life Technologies, Paisley, UK) and sterile MQ water for cDNA synthesis via reverse transcription.

Briefly, the presence of Kv4 channel mRNA in kisspeptin neurons was verified with single cell nested PCR reactions, in particular to detect Kv4.1, Kv4.2 and Kv4.3 gene expression. The cDNA was amplified with two consecutive PCRs, consisting of 40 cycles on the first round, 20 cycles on the second, and a 1:100 dilution of the product between first and second rounds, using two sets of primers (nested primers) for each gene of interest (See Table 1). The resulting product was analysed for appropriate size using 1.5% agarose gel electrophoresis. The *Hprt* housekeeping gene was used as an internal positive control to ensure that all cDNA conversions were successful. The pro‐opiomelanocortin (*POMC*) gene was used as an internal negative control to confirm that all tdTomato cells, and only tdTomato cells, expressed *Kiss1* mRNA. Additionally, sterile distilled water replacing cDNA was also used as a negative control.

**Table 1:** Primers used for nested RT‐PCR (see Methods)

Target	Sequence	Amplicon bp	Accession number
KV4.1 forward primer	ACCACACTTGGGTATGGAG	378	NM_008423.1
KV4.1 reverse primer	TGAACTCGTGACACGTAGTCTTCT		
KV4.2 forward primer	ACACTGGGGTATGGCGACA	379	NM_019697.3
KV4.2 reverse primer	AACTCATGGTTCGTGGTTTTCTC		
KV4.3 forward primer	CTACACTGGGATATGGAGACATGG	395	NM_001039347.1
KV4.3 reverse primer	GCTCATCAATAAACTCATGGTTAGTGG		
KISS 1 forward primer	GGAACTCGTTAATGCCTGGG	319	NM_178260.3
KISS 1 reverse primer	CTAGAAGCTCCCTGCCTTGG		
HPRT forward primer	ATGCCGACCCGCAGTC	563	J00423.1
HPRT reverse primer	GAATTTCAAATCCAACAAAGTCTGG		
Nested KV4.1 forward primer	TTGGGTCCATCTGCTCACTT	203	NM_008423.1
Nested KV4.1 reverse primer	GGCCCCCATTTTGCTTATAC		
Nested KV4.2 forward primer	ACCAAAACCAACGAGCAGAC	200	NM_019697.3
Nested KV4.2 reverse primer	TGGTGCTGTGTCTCAAAGCTG		
Nested KV4.3 forward primer	ACAAAAGAAGGCCCGCCT	130	NM_001039347.1
Nested KV4.3 reverse primer	CCTCTTCTGGGGTGCCC		
Nested Kiss forward primer	GAGAGCAAGCCTGGGTCTG	252	NM_178260.3
Nested Kiss reverse primer	AATCCACCTGCAGCCCA		
Nested HPRT forward primer	CGTGATTAGCGATGATGAACCA	486	J00423.1
Nested HPRT reverse primer	TTCACCAGCAAGCTTGCAAC		

### Data analysis and statistics

Data were analysed with custom Matlab scripts. All statistical values described in this work are shown as mean ± standard error of the mean (SEM), unless otherwise stated. In most experiments, adequate demonstration of normality of distribution was not possible, and so non‐parametric tests, like the Mann‐Whitney *U* test, were applied, except where stated.

## Results

### Kiss1^Arc^ neurons display an irregular firing pattern

Kisspeptin‐expressing neurons were distributed over a large portion of the arcuate nucleus, notably in the caudal region. These cells had very small somas, with long dendritic projections that were mainly oriented dorsally (Fig. 1 *A*), with a fairly high input resistance of 1.34 ± 0.69 GΩ, passive membrane time constant of 41.9 ± 18.55 ms, capacitance of 33.7 ± 13.68 pF and resting membrane potential of −67.4 ± 8.17 mV (mean ± SD, *n* = 49). Approximately 22% of cells (11/49) displayed spontaneous spikes at around 1 Hz (Fig. 1 *B*). Interestingly, when a step current injection was applied (from 2 to 10 s), most cells presented an irregular spiking pattern with sub‐threshold fluctuations, which was usually, but not always, more pronounced at lower firing frequencies (Fig. 1 *C*–*E*). Kiss1^Arc^ neurons resisted firing at high frequencies (Fig. 1 *F*), reaching a maximum of approximately 40 Hz, and exhibiting depolarisation block with strong current stimulation. Additionally, 29% (14/49) of the cells displayed a noticeable H current (Fig. 1 *G*) as described by others in Kiss1 neurons (Gottsch *et al*. 2011; Zhang *et al*. 2013). Although these different features illustrated the variation in electrophysiological properties of Kiss1^Arc^ neurons, no clear pattern emerged sufficient to suggest the existence of distinct populations.

### Kiss1^Arc^ neurons display sub‐threshold membrane fluctuations and persistent sodium currents

**Figure 2:**
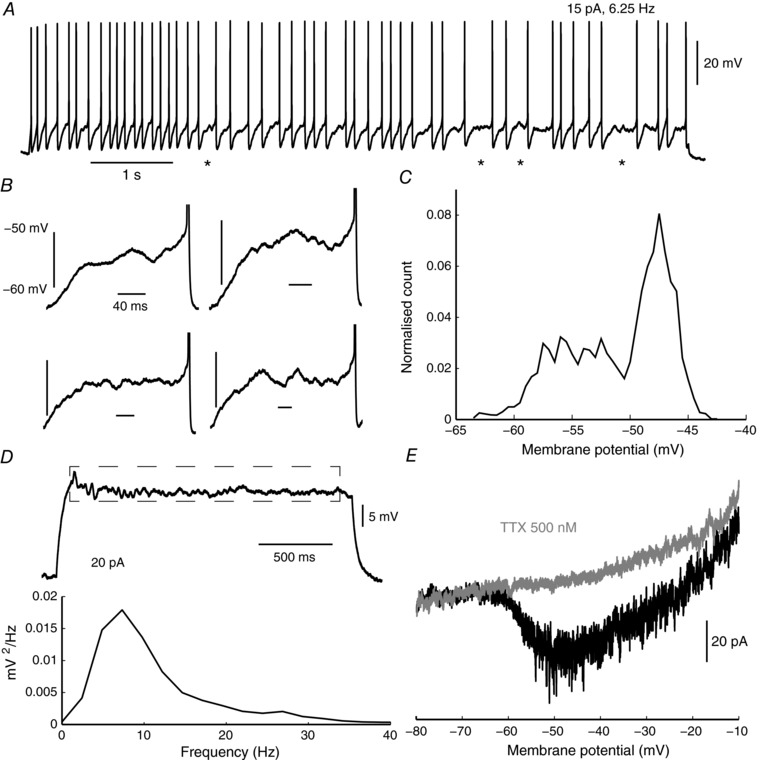
Irregular‐spiking kisspeptin neurons show APs separated by sub‐threshold voltage fluctuations *A* and *B*, detailed waveforms of noisy interspike intervals. Asterisk marks expanded examples. *C*, averaged membrane potential amplitude histogram of long ISIs (*n* = 9 neurons, each represented by 4 ISIs of around 100 ms) shows that fluctuations cover the range from −60 mV to −45 mV. *D*, power spectrum (below) of traces near the rheobase (example shown at top) reveals a broad distribution of low frequency fluctuations up to 30 Hz (*n* = 7, averaged and band‐pass filtered at 3–100 Hz). *E*, responses to slow depolarizing membrane potential ramps show salient non‐inactivating (persistent) sodium currents activating over the voltage range spanned by sub‐threshold oscillations. Inward peak current was 27.9 ± 2.6 pA at −50 mV, using a linear leak subtraction.

Irregular‐spiking kisspeptin neurons displayed highly variable interspike intervals (ISIs), characterised by noisy membrane potential fluctuations at the firing threshold, especially during longer intervals (Fig. 2 *A*–*C*), and with a noticeable low frequency component (up to 30 Hz, Fig. 2 *D*). As noisy sub‐threshold oscillations may perturb spike timing precision, they were assumed to be an important factor contributing to the generation of irregular firing. These fluctuations persisted throughout ISIs, with no apparent trend in amplitude over time during ISIs, suggesting that they were likely produced by non‐inactivating voltage‐dependent currents. Persistent sodium currents (NaP), which have already been described in Kiss1^RP3V^ neurons (Zhang *et al*. 2013), and are a likely candidate for involvement in the depolarizing phase of oscillations. We therefore sought to measure the level of NaP in Kiss1^Arc^ neurons.

In the presence of potassium and calcium channel blockers (TEA 2 mm, 4‐AP 2 mm and Cd^2+^ 200 μm), Kiss1^Arc^ neurons were subjected to a slowly depolarising voltage ramp (20 mV s^−1^), revealing a TTX‐sensitive inward current, activating at around −60 to −55 mV (Fig. 2 *E*, *n* = 9 total, *n* = 4 with TTX application), and with no apparent inactivation, characteristic of NaP. Based on this voltage range of activation it can be assumed that NaP is involved in the generation of sub‐threshold fluctuations. However, NaP is expressed in numerous types of neurons (Bean, 2007), and is not necessarily related to firing irregularity. We therefore looked for other sub‐threshold currents that could promote ISI noise, particularly currents that could interact with NaP.

### An A‐type current mediated by Kv4 is highly expressed in Kiss1^Arc^

**Figure 3:**
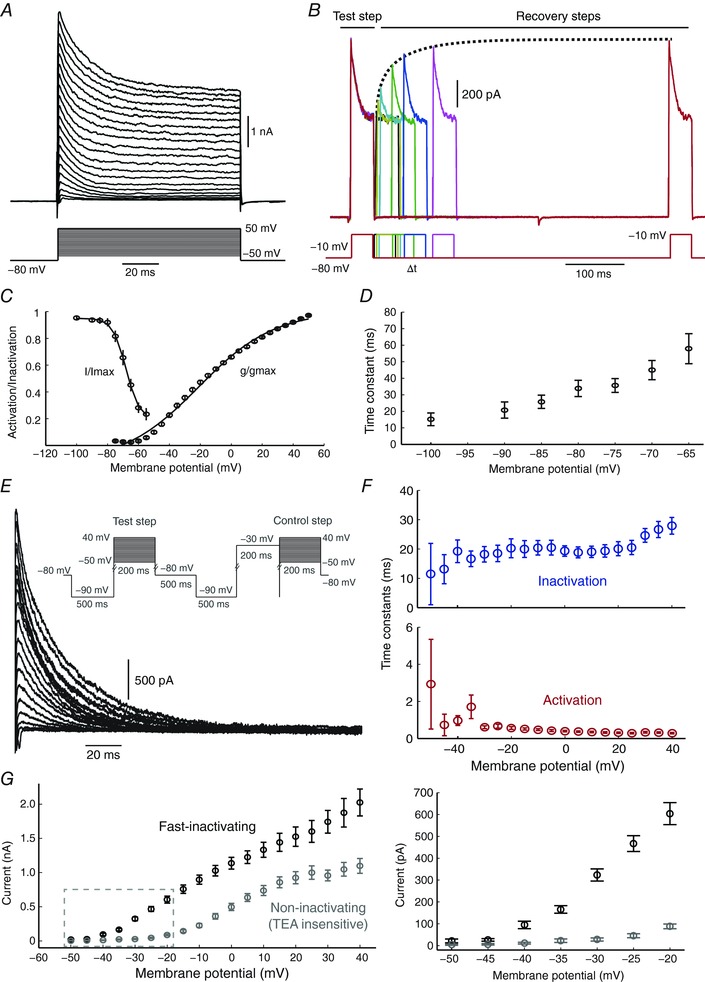
Kisspeptin neurons display a prominent A‐type current *A*, voltage steps from −50 to 50 mV reveal a fast‐inactivating outward current activating at around the spike threshold potential. *B*, illustration of the series of inactivating voltage steps that were used to characterise the kinetics of the A‐type current. *C* and *D*, data from *A* and *B* were used to reconstruct the steady‐state activation and inactivation curves (*C*), as well as the time constant for recovery of inactivation (*D*). *E*, inactivating pre‐pulses (inset) were applied in order to isolate the fast inactivating current component (in the presence of 5 mm TEA), which allowed characterisation of its activation and inactivation dynamics. For each voltage step, the isolated A‐type current was obtained by subtracting the control step (preceded by an inactivating step) from the test step. Kisspeptin neurons showed 16.2 ± 4.9 nS of peak conductance at 0 mV. *F*, the time constants for activation and inactivation were weakly voltage dependent. *G*, left: the peak current amplitude is dominated by fast‐inactivating currents, particularly at more negative membrane potentials. Right panel: zoomed region of *I–V* plot shown on the left, leak currents subtracted.

We attempted to characterise outward currents that were expressed in Kiss1^Arc^ neurons. When depolarising voltage steps were applied, these cells displayed a prominent A‐type K^+^ current, with a fast activating (≈3 ms time to peak) and fast inactivating (≈30 ms) component. Surprisingly, this was the dominant outward current type expressed in Kiss1^Arc^ (Fig. 3 *A*, *n* = 13). A‐type current is categorised as a sub‐threshold current (Bean, 2007), and since it was present in virtually all viable cells patched, we investigated its properties in more detail.

We used pre‐pulse steps (Burdakov & Ashcroft, 2002; Amarillo *et al*. 2008; Maffie *et al*. 2013) to isolate the A‐type current by subtraction, and measure its recovery from inactivation (Fig. 3 *B*, *n* = 17), as well as steady‐state activation and inactivation relationships (Fig. 3 *C*). Using the pre‐pulse method in the presence of 5 mm TEA also allowed accurate isolation of the A‐type current from other outward currents expressed in Kiss1^Arc^ neurons (Fig. 3 *E*–*G*, *n* = 16). We found that these cells expressed a total of 16.2 ± 4.9 nS peak conductance at 0 mV. The current activated at around −55 mV, and had a time constant for the exponential recovery of inactivation in the range 15–60 ms (weakly voltage‐dependent; Fig. 3 *F*). The peak outward current was dominated by the fast‐inactivating fraction, which comprised 70% of the total amplitude at 0 mV (1136 pA for A‐type current, and 496 pA for the remaining TEA‐resistant current, Fig. 3 *G*).

Kv4‐type channels are known to contribute substantially to the macroscopic A‐type current in many cell types, but in some cases, Kv1.4, Kv3.3 or Kv3.4 may also be involved, as they display similar fast‐inactivating properties. However, the kinetics of the A‐type current recorded in Kiss1^Arc^ neurons strongly suggest that it is mainly mediated by somatic Kv4 channels. Specifically, the similar activation and inactivation curves (Birnbaum *et al*. 2004), the fairly fast recovery from inactivation and the weak voltage dependence of its time course are all known properties of Kv4 channels. Participation of Kv1.4 can be discounted, as it has a time constant for the recovery from inactivation in the range of seconds (Wickenden *et al*. 1999).

**Figure 4:**
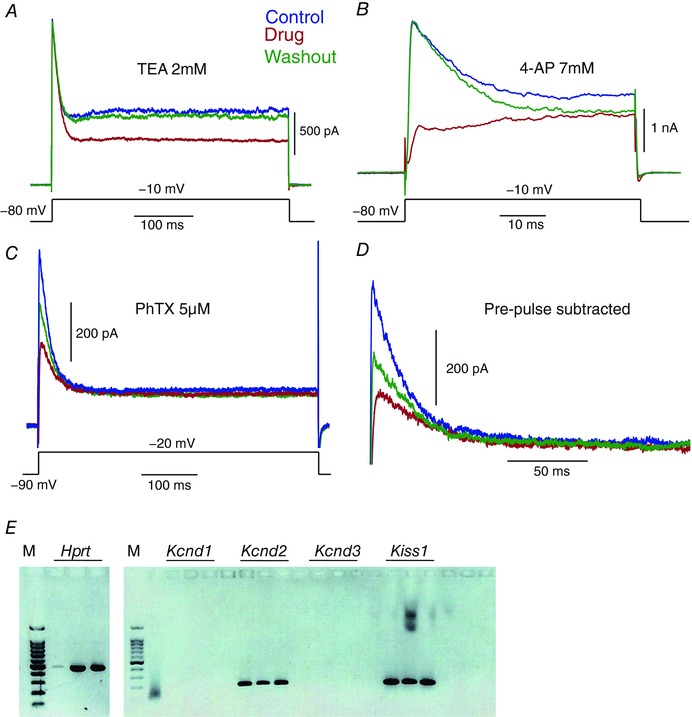
Pharmacological profile of A‐type current matches that of Kv4‐mediated current *A* and *B*, the fast‐inactivating current component was insensitive to TEA (*A*), but sensitive to 4‐AP (*B*). TEA blocked 27% of the maintained current. *C*, additionally, the current was sensitive to the specific Kv4.2 and Kv4.3 blocker phrixotoxin. *D*, phrixotoxin blocked 56% of the fast‐inactivating component isolated with the pre‐pulse protocol. *E*, representative single cell RT‐PCR transcript expression from a Kiss1 neuron. Cell contents were extracted from a single Kiss1 neuron and RNA converted into cDNA for nested PCR amplification of *Hprt*, *Kcnd1* (Kv4.1), *Kcnd2* (Kv4.2), *Kcnd3* (Kv4.3) and *Kiss1* transcripts. Each single cell cDNA was split into three experimental replicates for each transcript. RT‐PCR from a female ARC Kiss1 neuron is shown, but equivalent data was obtained for male Kiss1 neurons.

Pharmacological blockers further substantiated the presence of Kv4 current. The A‐type current was insensitive to the non‐specific Kv3 blocker TEA (Fig. 4 *A*, *n* = 5), but very sensitive to 4‐AP (Fig. 4 *B*, *n* = 5), which is a non‐specific Kv4 blocker. Lastly, we tested the specific Kv4.2/Kv4.3 blocker phrixotoxin (PhTX 5 μm; Fig. 4 *C*, *n* = 5) using local perfusion, and observed a mean reduction of 56% in the peak current (Fig. 4 *D*, *n* = 4), confirming the Kv4.2/4.3 identity of the A‐type current.

### Kv4 transcript expression in kisspeptin neurons at the single‐cell level

To corroborate the electrophysiological data, expression of the Kv4 potassium channel subunits Kv4.1, 4.2 and 4.3 was examined by RT‐PCR following harvesting of cytoplasm from single Kiss1^Arc^ cells (Fig. 4 *E*). A total of 15 arcuate tdTomato/kisspeptin cells were screened for *Kiss1*, *Hprt* and Kv4 subtype transcripts, and we observed that Kv4.2 expression predominated: 86% of Kiss1^Arc^ neurons were positive for this Kv4 subtype. In contrast, only 9% of the cells expressed Kv4.3, while no Kv4.1 expression was found. The predominant expression of Kv4.2 suggested that the prominent A‐type current was mainly generated by this channel subtype.

### Spiking irregularity is correlated with the presence of Kv4 conductance in Kiss1^Arc^ neurons

Kiss1^Arc^ neurons displayed an abundant Kv4‐type conductance (*g*_Kv4_)_,_ which was presumed to affect their firing properties, as it activated at fairly low membrane potentials, and would thus be expected to influence the trajectory of membrane potential as it approaches threshold. We therefore anticipated that Kv4 current could affect the spiking irregularity observed in Kiss1^Arc^ cells, in analogy to its role in irregular‐spiking cortical interneurons, as shown previously (Mendonça *et al*. 2016).

**Figure 5:**
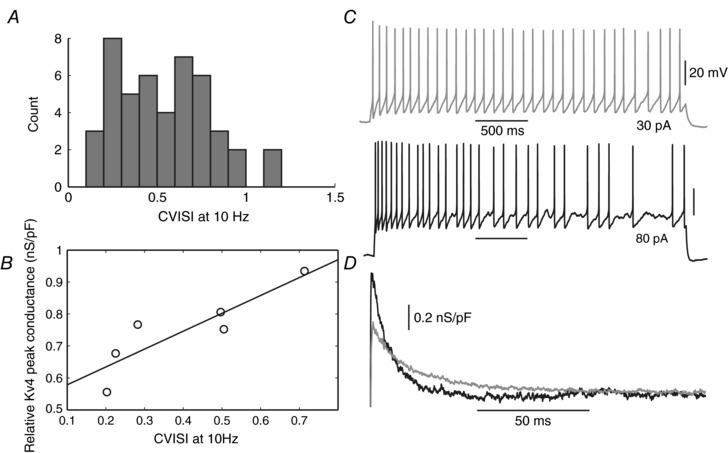
Native Kv4‐type conductance is correlated with the intrinsic spiking irregularity of kisspeptin neurons *A*, the population of kisspeptin neurons shows a broad distribution of spiking irregularity (*n* = 47 cells). *B*, in some cells with low series resistance (<20 MΩ) permitting adequate voltage clamp, both spiking irregularity and Kv4 current amplitude were measured. The coefficient of interspike interval (CVISI) at 7–13 Hz firing frequency was then estimated, while the peak Kv4 conductance was normalised to the capacitance of each cell. Pearson correlation *P* value: 0.019, *r* value = 0.88, *n* = 6. *C*, example segments of firing from a regular‐spiking cell (grey) contrasting with an irregular‐spiking cell (black). *D*, corresponding isolated Kv4 conductance waveforms for voltage steps from −90 to −20 mV obtained with the pre‐pulse protocol.

In order to test this possibility, we first quantified the irregularity of kisspeptin neuron firing by estimating the coefficient of the interspike interval (CVISI) at 10 Hz (CVISI_10_, from 7 to 13 Hz). We observed that these cells were not homogeneous in this regard. Instead, they displayed a broad distribution in their spiking irregularity (Fig. 5 *A*, *n* = 47). While some cells fired with a fairly regular pattern (CVISI_10_ < 0.2), most cells displayed an irregular‐firing pattern, ranging from relatively mild (≈0.3) to very high (>0.7) spiking irregularity.

The broad distribution of spiking irregularity found in Kiss1^Arc^ neurons suggests that these cells might naturally express ion channels in different proportions, resulting in different firing patterns. We were particularly interested to test whether heterogeneity of spiking irregularity was related to distinct Kv4 expression in each neuron. Therefore, in some cells, we assessed both CVISI_10_ and pre‐pulse isolated *g*_Kv4_, which was possible due to their relative small soma size (space‐clamp without ‘escaping’ sodium currents). When *g*_Kv4_ was normalised by the capacitance of each cell (proportional to their membrane area), we found a clear correlation between firing irregularity and *g*_Kv4_ density (Fig. 5 *B*–*D*, compare *C* and *D*). These findings suggest that the firing variability of Kiss1^Arc^ neurons could be determined by *g*_Kv4_.

### Artificial injection of Kv4 conductance modulates spiking irregularity and excitability

As *g*_Kv4_ was apparently associated with the intrinsic spiking irregularity of some Kiss1^Arc^ neurons, the dynamic‐clamp conductance injection technique was used to investigate this link further. This method allows injection of any mathematically‐described voltage‐dependent conductance in a whole‐cell current‐clamp recording (Robinson & Kawai, 1993; Sharp *et al*. 1993b; Robinson, 2013), allowing us to add or subtract controlled levels of *g*_Kv4_ conductance, and test its effect on firing irregularity.

**Figure 6:**
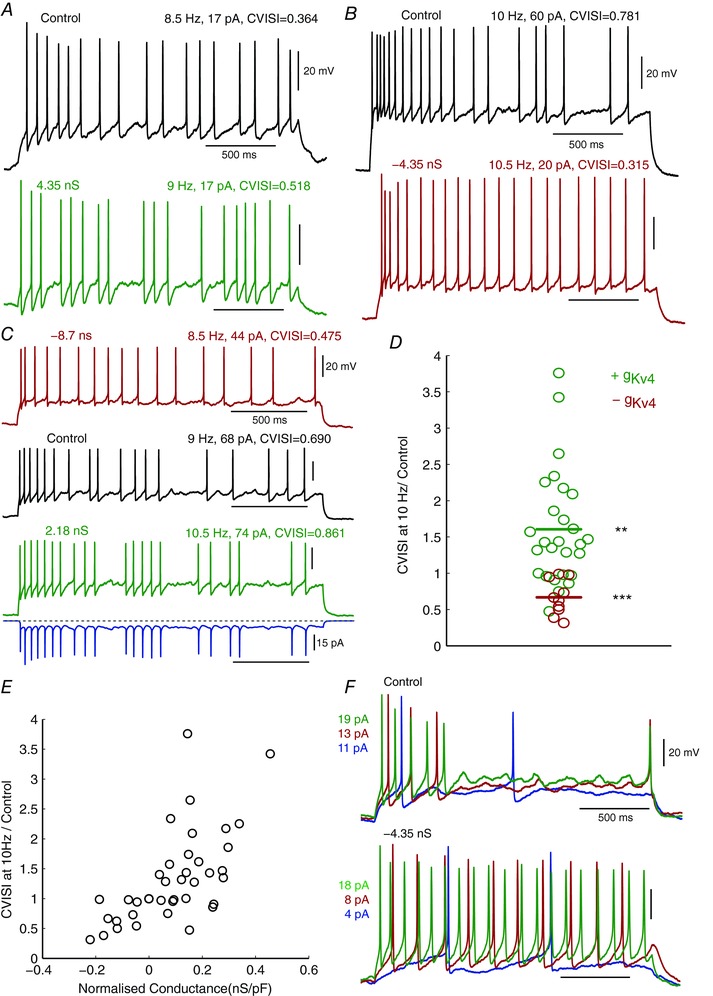
Injection of synthetic Kv4‐type conductance effectively controls spiking irregularity *A*–*C*, examples of cells subjected to *g*_Kv4_ dynamic clamp, comparing responses with similar firing frequencies. In example shown in *C*, the bottom blue trace shows the injected Kv4 current (inward current downwards) during the injection of +2.18 nS conductance (green trace). *D*, summary of CVISI_10_ of all cells subjected to *g*_Kv4_ modulation. Mann‐Whitney test, *P* value = 0.0012 and 6.38 × 10^−5^ for positive and negative *g*_Kv4_ injection. *E*, a positive correlation was found between the change in spiking irregularity and the amount of *g*_Kv4_ injected (normalised by each cell's capacitance). Pearson's correlation *r* value = 0.655, *P* value = 7.8 × 10^−8^. *F*, in some strongly adapting cells, it was observed that negative *g*_Kv4_ injection produced a more regular firing, which could reach higher frequencies with similar or lower current stimulation than control.

When *g*_Kv4_ was altered in Kiss1^Arc^ neurons, we saw a striking effect on their intrinsic spiking irregularity. When positive *g*_Kv4_ was injected in mildly irregular‐firing cells, we observed a clear increase in interspike variability, which was normally associated with the presence of prominent noisy sub‐threshold ‘plateaus’ (Fig. 6 *A* and *C*). In contrast, negative *g*_Kv4_ injection in very irregular‐firing cells resulted in more regularly distributed action potentials (Fig. 6 *B* and *C*). A clear correlation was found between CVISI_10_ and the normalised level of *g*_Kv4_ injected (Fig. 6 *E*, *n* = 17).

However, *g*_Kv4_ modulation did not only impact how irregularly Kiss1^Arc^ neurons fired, but also created a distinct effect on spiking adaptation. Some neurons displayed a significant intrinsic decrease in the firing frequency throughout the constant stimulation, which was usually observed in very irregular‐spiking cells. When *g*_Kv4_ was reduced in these neurons, they not only produced a more regularised spiking pattern, but also fired steadily at higher frequencies at lower levels of stimulus current (Fig. 6 *F*).

## Discussion

Here, we have characterised the electrophysiological properties of arcuate nucleus kisspeptin neurons from a novel *Kiss1*‐Cre:tdTomato transgenic mouse line, providing an insight into the biophysical mechanisms governing their action potential generation. Some of the electrophysiological properties shown here for kisspeptin neurons have already been described by others in different mouse strains, such as the high input resistance, the expression of H‐current, persistent sodium current, and spontaneous spiking (Kelly *et al*. 2013; Zhang *et al*. 2013; Piet *et al*. 2014). In this study, we find that these cells also express a strong A‐type potassium current. Using pharmacological blockers and single cell RT‐PCR, we demonstrated that this current is mainly mediated by Kv4‐type channels (likely predominantly Kv4.2). Furthermore, we have highlighted the role that this current plays in shaping the firing pattern of *Kiss1* ^Arc^ neurons. By interacting with subthreshold inward currents (like NaP), these channels can create interspike voltage fluctuations, which affect the timing of spikes and diversify their firing patterns. Using dynamic‐clamp conductance injection, we have shown that modulation of *g*_Kv4_ not only changed their firing irregularity, but controlled their firing frequency in response to a constant stimulus. These findings are especially relevant when considering that kisspeptin neurons are normally stimulated tonically by neuropeptides like neurokinin‐B (NKB; De Croft *et al*. 2013).

Since its early characterisation, A‐type current, commonly but not exclusively carried through Kv4 ion channels, has mainly been associated with enabling low‐frequency, regular firing activity (Connor & Stevens, 1971). Here, by contrast, we confirm that, under the right conditions, *g*_Kv4_ can contribute to sub‐threshold fluctuations and cause an increased irregularity in action potential timing. The mechanism we report here is strikingly similar to what we have observed recently in irregular‐firing interneurons (Mendonça *et al*. 2016), where the same current types (A‐type and NaP) have been shown to create an almost identical effect on spiking irregularity. However, although we show that Kiss1^Arc^ neurons also display the same components, we cannot exclude the possibility that other subthreshold currents (e.g. T‐type Ca^2+^ currents) contribute significantly to this mechanism. Using computational modelling, the dynamical basis of increased irregular firing in cortical interneurons produced by addition of *g*_Kv4_ was made clear: both unstable oscillations and slow dynamics in the interspike interval emerge as *g*_Kv4_ is increased, through its dynamical interactions with the sodium current, including NaP (Mendonça *et al*. 2016). It is interesting that cells embedded in completely different physiological contexts, in different brain regions, seem to use a similar control of firing, which may be viewed as a conserved biophysical mechanism for generating intrinsic spiking irregularity for different purposes in the brain.

Even though it is plausible to draw close comparisons between the firing dynamics of irregular‐spiking cortical interneurons and Kiss1^Arc^ neurons, some distinctions need to be made. Most importantly, the currents required to generate spiking irregularity were present in different proportions in these two cell types. While irregular‐spiking interneurons displayed a large NaP current (80.9 pA at −50 mV, or 1.51 pA/pF normalised to the overall mean of the capacitance), kisspeptin neurons displayed a lower level (27.9 pA at −50 mV, or 0.83 pA/pF). In contrast, while irregular‐spiking cells expressed sufficient Kv4 conductance to fire irregularly (22.3 nS peak at 0 mV, or 0.42 nS/pF), it was even higher in kisspeptin neurons, representing one of the dominant K^+^ conductance fractions (16.2 nS peak at 0 mV, or 0.48 nS/pF). Reflecting the relative dominance of *g*_Kv4_, kisspeptin neurons were more responsive to its modulation. A reduction of *g*_Kv4_ translated into a significant decrease in one of the most important outward conductances in these cells, which as expected, markedly increased their excitability, especially in irregular‐spiking cells with strong firing adaptation. We propose that the level of expression of functional Kv4 channels, in addition to modulating firing irregularity, acts as a ‘gain controller’ of kisspeptin neurons, determining the size of responses to their synaptic inputs.

A similar phenomenon, where A‐type current acts as a ‘gain controller’ has been proposed in orexin‐expressing neurons in the lateral hypothalamus (Burdakov *et al*. 2004), where spontaneously firing cells required less hyperpolarising current to reduce firing, when they expressed larger A‐type conductances, implying that A‐type current raises the effective gain of the firing frequency–stimulus current relationship. Interestingly, in addition to orexin and kisspeptin neurons, there are reports of several other cell types in the hypothalamus displaying A‐type currents (Burdakov & Ashcroft, 2002; Zhang & van den Pol, 2015), suggesting that hypothalamic neurons may use A‐type conductance modulation (or specifically Kv4 conductance) as a common strategy to control their excitability.

The surprising heterogeneity of spiking irregularity found in kisspeptin neurons, associated with different levels of *g*_Kv4_ expression, suggests a natural fine tuning in these cells, which due to temporary or permanent adjustment of Kv4 channel kinetics, might allow each Kiss1^Arc^ neuron to have distinctive influences on GnRH neurons and/or other kisspeptin neurons. As the frequency and temporal structure of firing can determine the differential release of neurotransmitters (Dutton & Dyball, 1979; Liu *et al*. 2011; Schöne *et al*. 2014; Qiu *et al*. 2016), the level of Kv4 expressed in each cell can favour the release of different neuropeptides or amino acid neurotransmitters.

However, it is still unclear if these cells can actively change their firing properties by altering the total level of somatic *g*_Kv4_. The gating kinetics of Kv4 channels are known to be modulated by accessory proteins (K^+^ channels interacting proteins, KChIPs) and membrane lipids (Birnbaum *et al*. 2004; Oliver *et al*. 2004), while some neuromodulators, like endocannabinoids and cholecystokinin, have been shown to modify Kv4‐type current amplitude (Burdakov & Ashcroft, 2002; Amorós *et al*. 2010). We tested the effect of perfusing three different neuromodulators, neurokinin‐B (10 μm, *n* = 7) and dynorphin‐A (50 μm, *n* = 5), known to affect the excitability of kisspeptin neurons, and cholecystokinin (100 pm, *n* = 3; 40 pm, *n* = 3; 50 pm, *n* = 2; 100 nm, *n* = 1) which has been reported to increase A‐type conductance in arcuate cells (Burdakov & Ashcroft, 2002). These peptides produced no noticeable acute effects on *g*_Kv4_ in kisspeptin neurons (data not shown), but it is still possible that other neuromodulators are capable of doing so. It is also possible that *g*_Kv4_ is not subject to significant neuromodulation in Kiss1^Arc^ neurons, but that variation in *g_Kv4_* density may be determined by connectivity and particular role of a neuron within the circuit, and by stochastic gene expression and trafficking of the channel protein.

In summary, we propose that the density of Kv4, predominantly Kv4.2, channels in arcuate kisspeptin neurons is an important factor that regulates their firing pattern and excitation of GnRH neurons and other kisspeptin neurons. When Kv4 is strongly expressed, Kiss1^ARc^ neurons fire at very low frequency and asynchronously, likely contributing to the release of amino‐acid neurotransmitters. However, when Kv4 channels are downregulated or inhibited, these cells are capable of delivering a more reliable stimulation at a higher firing frequency, favouring the release of neuropeptides and effectively recruiting other kisspeptin neurons and GnRH neurons.

## Additional information

### Competing interests and funding

There were no competing interests.

### Author contributions

All the authors contributed to planning and design of the study. Electrophysiology experiments and analysis were carried out by P.M. GM mouse breeding, immunostaining, confocal imaging and single‐cell RT‐PCR were carried out by V.K., S.H.‐Y. and P.M. P.M., W.C. and H.R. wrote the manuscript. All authors have approved the final version of the manuscript and agree to be accountable for all aspects of the work. All persons designated as authors qualify for authorship, and all those who qualify for authorship are listed.

### Funding

Funding was contributed by Coordenação de Aperfeiçoamento de Pessoal de Nível Superior (PM), and the BBSRC (Grant No. BB/K003178/1).
